# Adaptation of Postural Sway in a Standing Position during Tilted Video Viewing Using Virtual Reality: A Comparison between Younger and Older Adults

**DOI:** 10.3390/s24092718

**Published:** 2024-04-24

**Authors:** Tsubasa Tashiro, Noriaki Maeda, Takeru Abekura, Rami Mizuta, Yui Terao, Satoshi Arima, Satoshi Onoue, Yukio Urabe

**Affiliations:** Department of Sports Rehabilitation, Graduate School of Biomedical and Health Sciences, Hiroshima University, 1-2-3 Kasumi, Minami-ku, Hiroshima 734-8553, Japan; tsubasatashiro716@hiroshima-u.ac.jp (T.T.); takeruabekura1124@hiroshima-u.ac.jp (T.A.); rami-mizuta@hiroshima-u.ac.jp (R.M.); satoshi-arima4646@hiroshima-u.ac.jp (S.A.); satoshi-onoue@hiroshima-u.ac.jp (S.O.)

**Keywords:** virtual reality, older adults, center of pressure, age difference, sex difference

## Abstract

This study aimed to investigate the effects of wearing virtual reality (VR) with a head-mounted display (HMD) on body sway in younger and older adults. A standing posture with eyes open without an HMD constituted the control condition. Wearing an HMD and viewing a 30°-tilt image and a 60°-tilt image in a resting standing position were the experimental conditions. Measurements were made using a force plate. All conditions were performed three times each and included the X-axis trajectory length (mm), Y-axis trajectory length (mm), total trajectory length (mm), trajectory length per unit time (mm/s), outer peripheral area (mm^2^), and rectangular area (mm^2^). The results showed a significant interaction between generation and condition in Y-axis trajectory length (mm) and total trajectory length (mm), with an increased body center-of-gravity sway during the viewing of tilted VR images in older adults than in younger adults in both sexes. The results of this study show that body sway can be induced by visual stimulation alone with VR without movement, suggesting the possibility of providing safe and simple balance training to older adults.

## 1. Introduction

Fractures due to falls in older adults can significantly limit their daily lives, making it a global challenge [1]. Balance improvement is an important factor in fall prevention [2]. Exercises using traditional balance discs or balls have demonstrated improvements in balance capabilities. Such training activates the postural control responses required to maintain stability on an unstable surface, where the body’s center of gravity sways forward, backward, and sideways on the stable board. Consequently, the body’s ability to balance external interference is enhanced [3]. However, for many older adults, maintaining a static standing posture may be challenging, and balance exercises that are too difficult may diminish the learning effect [4]. In contrast, if the difficulty level of balance exercises is too easy, the advantages of learning are insufficient [5]. Therefore, there is a need to develop postural control exercises that allow for gradual adjustment of difficulty levels and maintain participant engagement and enjoyment. 

The virtual reality (VR) technology uses a head-mounted display (HMD) to present 360° images by visually placing users in a virtual space. One of the attractive features of VR is that it can provide motor-related stimulation to the brain with relative safety [6]. Promoting physical activity (PA) through exercise or interactive virtual games using VR has emerged as a potential technology for improving balance, posture, gait, and overall health in older adults [7,8]. A systematic review has also shown that training in VR using an HMD could be useful for fall prevention and postural control in older people; however, there are concerns that the research in older people is of poor quality due to challenges in ensuring safety and a high risk of bias [9]. Urabe et al. used an HMD to demonstrate fluctuations in the center-of-gravity sway during a static standing posture in younger adults [10]. This study was a milestone compared to conventional balance training in terms of safety, which induces center-of-gravity sway during stationary standing without physical movement but was limited to a young cohort. Therefore, it is necessary to examine whether similar results apply to older adults who are at high risk of falling in their daily lives. The 2020 Scoping Review reported that attempts to use HMD to improve balance in older adults have great potential [11]; however, there are still few reports on their impact.

In addition, whether changes in postural balance are influenced by sex has been debated. According to recent sex-difference analyses in older adults, the decline in static balance capacity appears to be greater in men than in women [12,13]. For example, older men demonstrate decreased accuracy in center-of-gravity positioning, especially with somatosensory and visual deprivations, which are associated with altered postural control strategies [14]. However, to the best of our knowledge, the effects of VR interventions on sex differences in postural control have not yet been examined. Previous studies focusing on similar scenarios to improve ADLs in older people through the application of pioneering devices have increased in recent years [15,16]. Our research has the potential to serve as a basic database for providing balance training that is more familiar and easier to use for older people.

The purpose of this study was to investigate the effects of viewing tilted VR images while wearing HMD on the displacement of the body’s center of gravity while standing in younger and older adults. A scene without an HMD (real world) was set as a control condition to confirm the effect of wearing an HMD on the displacement of the body’s center of gravity. A sex comparison was also conducted, with the hypothesis that men would show a more pronounced center of gravity sway than women.

## 2. Materials and Methods

### 2.1. Study Design and Participants

This was an observational cross-sectional study. Here, 20 younger adults (10 women) aged 18–29 years and 34 older adults (24 women) aged >65 years participated in the study. Younger adults were recruited from among those who had read research posters at Hiroshima University and were willing to participate in the study. To recruit older participants, we reached out to representatives of welfare facilities in Hiroshima Prefecture to request research cooperation. We identified exercise communities in different regions of Hiroshima. Among them, those who expressed a willingness to cooperate were included in the study. All older participants belonging to these exercise communities were attending a weekly gymnastics class. The exclusion criteria were as follows: (1) visual impairment (total blindness and low vision); (2) diseases that may affect balance function (Parkinson’s disease, vertigo, Meniere’s disease, and dysfunction of the semicircular canal or inner ear); (3) difficulty viewing VR images due to poor health or physical pain on the day of measurement; (4) sensitivity to light stimuli; and (5) history of image sickness.

The sample size was calculated using G* power 3.1, with a one-way repeated-measures (ANOVA) test (effect size = 0.25 [medium], alpha error = 0.05, power = 0.80, number of groups = 6, number of measurements = 3, Corr among rep measures = 0.5, Nonsphericity correction ε = 1) [17]. Due to its novelty and lack of precedent, average figures were applied to the power analysis. Based on this test, a minimum of 54 participants was required for the study.

This study conformed to the guidelines of the Declaration of Helsinki and was approved by the Ethics Committee for Epidemiology of Hiroshima University (E-2299). Informed consent was obtained from all study subjects.

### 2.2. Assessment of Physical Activity

Physical activity and sedentary time were assessed using the International Physical Activity Questionnaire–Short Form (IPAQ-SF) [18]. We assessed vigorous PA, moderate PA, and average walking time per week, and total PA was subsequently calculated (Mets*mins/week). The participants were also questioned about their sedentary time on weekdays.

### 2.3. Study Setting

**Figure 1:**
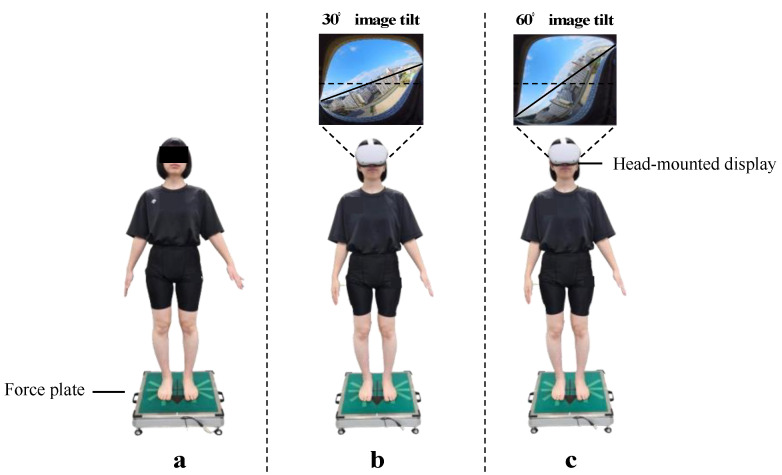
Three conditions were used in this study: (**a**) control condition without a head-mounted display; (**b**) VR30° condition while viewing a 30°-tilted image with a head-mounted display; and (**c**) VR30° condition while viewing a 60°-tilted image with a head-mounted display.

All assessments were conducted under three conditions: a quiet environment where no additional external information such as visual or auditory stimuli interferes; a resting standing posture with eyes open without an HMD (Oculus Quest 2, Meta Inc., California, United States) (control condition); a resting standing posture while wearing an HMD and viewing a VR image tilted by 30° (VR30°) gradually; and a resting standing posture while wearing an HMD and viewing a VR image that gradually tilts 60° (VR60°) (Figure 1). For the tilt of the VR image, an image of an outdoor landscape seen from a room on the 9th floor of Hiroshima University was captured beforehand with a 360° camera (Key Mission 360, Nikon, Tokyo, Japan). In the control condition, the participants were instructed to stand facing a blank wall and stare at a target placed 2 m in front of them at eye level. In the VR30° and VR60° conditions, visual input was provided only from the glasses, without any other peripheral visual input, so that the gaze was maintained on the VR screen. The speed of image tilt was 3°/s for VR30° and 6°/s for VR60°. Each condition was performed every alternate day in a randomly assigned order using a computerized random number. 

### 2.4. Assessment of Center of Gravity

A force plate (T.K.K. 5810, Takei Measuring Instruments, Inc., Niigata, Japan) was used to assess the center of gravity. The force plate sampling frequency was 100 Hz. The center of gravity was assessed simultaneously during the implementation of each condition. The participants maintained a standing posture on a force plate with both feet shoulder-width apart and both arms on the side of the body. To ensure safety, two assistants were placed on either side of the participant during the measurement.

On the force plate, the following six parameters were measured: X-axis trajectory length (mm), Y-axis trajectory length (mm), total trajectory length (mm), trajectory length per unit time (mm/s), outer peripheral area (mm^2^), and rectangular area (mm^2^) [19]. Participants performed three trials for each condition. The X-axis trajectory length (in mm) denotes the distance covered horizontally, whereas the Y-axis trajectory length (in mm) represents the vertical distance covered by a point or object. The total trajectory length (mm) combines both the X- and Y-axis movements. The trajectory length per unit time (mm/s) was used to measure the speed of movement. The outer peripheral area (mm^2^) refers to the region enclosed by the outermost points, and the rectangular area (mm^2^) signifies the space enclosed by the outer boundary of an object or trajectory.

### 2.5. Statistical Analysis

Statistical analysis was performed using SPSS software (version 27.0; SPSS Japan Inc., Tokyo, Japan). The normality of all variables was confirmed using the Shapiro–Wilk test. To compare the physical characteristics and physical activities between the younger and older adults, either an unpaired *t*-test or Mann–Whitney U test was employed. To investigate the intersection effect of COP movement, a two-way repeated-measures analysis of variance was conducted with the age group (younger and older adults) as the between-subject factor and condition (control, VR30°, VR60°) as the within-subject factor. If interaction effects were observed, post-hoc tests were conducted using unpaired t-tests for Generation and the Bonferroni test for condition. A Mann–Whitney U test was performed to compare sex differences in the COP movement. The significance level was set at 5%.

## 3. Results

In this study, none of the participants withdrew from the VR intervention because of simulator sickness. Regarding the participants’ descriptive statistics (Table 1), significant differences existed in age, height, and weight between the younger and older adult groups. In terms of PA levels, significant between-group differences were observed in total PA (Mets*mins/week), vigorous PA (Mets*mins/week), moderate PA (Mets*mins/week), walking (Mets*mins/week), and sedentary time (min/day). Regarding the results of center of pressure (COP) movement (Table 2), significant interaction effects of generation and condition were observed for the Y-axis trajectory length (mm) (F = 3.436, *p* = 0.036) and total trajectory length (mm) (F = 7.878, *p* < 0.001). The participant generation significantly influenced the Y-axis and total trajectory lengths (*p* = 0.045 and *p* < 0.001, respectively). This condition also had a significant effect on the Y-axis and total trajectory length (*p* < 0.001). Post-hoc tests revealed that the Y-axis trajectory length was significantly greater in younger adults at −5.58 [−7.12 to −1.14] mm for VR60° than the 2.05 [−5.20 to 5.33] mm in the control (*p* < 0.05). In the VR60° condition, there was a significant difference in the Y-axis trajectory length between −5.58 [−7.12 to −1.14] in younger adults and 1.08 [−4.08 to 3.91] in older adults (*p* < 0.05). In terms of total trajectory length, the values of 157.45 [125.53–249.02] for the VR30° condition and 188.85 [112.75–283.36] for the VR60° condition were both significantly greater than those for the control in older adults, whereas no differences between conditions were observed in younger adults (*p* < 0.05, respectively). In generational comparisons, the total trajectory length was greater for the older adults than for the younger adults under all conditions (*p* < 0.001, respectively).

A comparison of COP movements between men and women is presented in Table 3. For the X-axis trajectory length, women had significantly larger values than men in the VR30° and VR60° conditions in younger adults, whereas men had larger values than women among older adults (*p* = 0.043 and *p* = 0.015, respectively). Focusing on women, significant differences were observed in the X-axis trajectory length between younger and older adults in the VR30° and VR60° conditions (*p* = 0.002 and *p* = 0.001, respectively). In terms of the Y-axis trajectory length, men showed significantly greater values than women at VR60° (*p* = 0.010). In women, there was a significant difference in the Y-axis trajectory length between younger and older adults in the VR60° condition (*p* = 0.023). For total trajectory length, older adults showed significantly greater results than younger adults in the control, VR30°, and VR60° conditions in men (*p* = 0.035, *p* < 0.001, and *p* = 0.002, respectively) and women (*p* = 0.010, *p* < 0.001, and *p* < 0.001, respectively). The trajectory length per unit time in older men (*p* = 0.035, *p* < 0.001, and *p* = 0.002, respectively) and women (*p* = 0.006, *p* = 0.004, and *p* < 0.001, respectively) under the control, VR30°, and VR60° conditions were significantly greater than those in younger adults. When examining the outer peripheral area, older adults showed larger values than younger adults in women under the VR30° and VR60° conditions (*p* = 0.046 and *p* = 0.013, respectively).

## 4. Discussion

As the world population ages, preventing the negative consequences of falls has become a critical public health mission [1]. This study aimed to investigate how the tilt of images displayed through HMD affected the displacement of the center of mass in both younger and older individuals. The results revealed that older adults experienced greater displacement of their center of mass due to the tilt of the VR images than younger individuals. Furthermore, the tilt of the VR images at 60° had a greater impact on the COP than that at 30°. To the best of our knowledge, this is the first study to capture the displacement of the center of mass solely through the tilt of VR images without accompanying physical movements using HMD technology.

The most important finding of this study was that the total trajectory length was greater in the VR condition for older adults than for younger adults. Balance impairments are known to be proportional to aging, and a similar trend was observed in our validation using VR. A cross-sectional validation study by Imaoka et al. also reported that postural movements were greater in older adults than in younger adults after viewing VR [20]. There are two main explanations for the differences in postural control between younger and older adults. One explanation for this is the effect of aging on physical function. A 2015 systematic review showed a relationship between age-related muscle weakness and a reduced ability to control posture [21]. Our results also showed that in the control condition without VR, older adults had a greater total trajectory length than younger adults. Another possible explanation is the relationship between aging and visual feedback. A previous study using optic flow stimulation found that adaptation to visual stimuli in the standing position was longer in older adults than in younger adults [22]. Another study on optic flow stimulation during treadmill walking found that the visual attention required to process visual flow during walking was delayed in older adults than in younger adults [23]. Thus, these and other declines in perceptual inhibition in older adults could contribute to an increased body center-of-gravity sway in response to slowly changing VR image tilts.

According to previous studies, men have been reported to exhibit greater oscillations in their body’s center of mass, which may stem from differences in anthropometric characteristics, such as height, between the sexes [12,24]. With increased height, the center of mass of the body tends to increase, thereby necessitating more postural control strategies in men than in women. Although men tend to be taller than women from puberty onward, contrasting results were obtained in our study’s younger cohort, in which women showed greater oscillations in their body’s center of mass. An individual’s sense of immersion when viewing a tilted VR image is notable. The Simulator Sickness Questionnaire 2 (SSQ) score, which is often used in VR research to assess sickness when using HMD, has shown inconsistent results for sex differences. In some studies, women are more susceptible to VR sickness than men, reporting higher SSQ scores [25,26,27]. However, Lawson (2014), based on a review of 46 previous studies, found that claims of sex differences in VR sickness were inconclusive [28]. These may explain the discrepancy between men and women in the X-axis trajectory lengths obtained in this study. 

The failure to measure SSQ to assess VR immersion is a limitation of this study. Various factors influence sex differences between immersions when using HMD, such as women’s hormone levels [29] and history of motion sickness [30], which need to be considered to examine sex differences in future VR-induced sway of the body center of gravity.

The second limitation is that the data did not encompass a wide range of age groups. Third, an assessment of immersiveness in the VR intervention was not included. Finally, regarding the interpretation of postural sway through the HMD, evaluations of parameters such as neuromuscular, sensory, and proprioceptive receptors were not performed.

The present study indicates that watching a tilting VR movie is sufficient to induce center-of-gravity sway in elderly people. Therefore, we suggest that viewing a tilting VR movie can be used in the future as a balance exercise that makes it easy to adjust the level of difficulty. However, to make it a more effective training method, future studies should consider adopting other latest technologies. For example, in the field of artificial intelligence, it is possible to estimate human posture online [31,32], and balance information during VR movie viewing can be feedbacked to make posture control exercises for the elderly by viewing tilting VR movies even more effective.

## 5. Conclusions

In conclusion, wearing VR head-mounted goggles and viewing tilting VR images increased body center-of-gravity sway more in older adults than in younger adults during standing posture in both sexes. Our study suggests that the use of HMD may be a safe and convenient way to provide balance training to older adults in the future, without physical movements.

## Figures and Tables

**Table 1:** Descriptive statistics of participants.

	Younger Adults (*n* = 20)	Older Adults (*n* = 34)	*p*-Value
Women (%)	10 (50.0%)	24 (70.6%)	-
Age (years)	23.15 ± 1.04	75.56 ± 5.26	**<0.001** ^a^
Height (cm)	164.02 ± 6.86	157.05 ± 10.79	**<0.001** ^a^
Body weight (kg)	58.99 ± 8.52	54.78 ± 11.08	**<0.001** ^a^
Body mass index (kg/m^2^)	21.87 ± 2.43	22.05 ± 2.95	0.557 ^a^
IPAQ-SF			
Total PA (Mets*mins/week)	1476.00 [770.25–2351.25]	832.00 [396.00–1805.25]	**<0.001** ^b^
Vigorous PA (Mets*mins/week)	720.00 [0.00–960.00]	0.00 [0.00–0.00]	**<0.001** ^b^
Moderate PA (Mets*mins/week)	420.00 [20.00–480.00]	0.00 [0.00–480.00]	**<0.001** ^b^
Walking (Mets*mins/week)	297.00 [0.00–594.00]	627.00 [321.750–1188.00]	**<0.001** ^b^
Sedentary Time (min/day)	600.00 [600.00–780.00]	300.00 [180.00–555.00]	**<0.001** ^b^

Data are expressed as means ± standard deviation or medians [interquartile range], ^a^ means *p*-value for paired *t*-test, ^b^ means *p*-value for Mann–Whitney U test, IPAQ-SF International Physical Activity Questionnaire.

**Table 2:** Results of two-way analysis of the center of pressure movement.

		Generation		Interaction Effect (Generation × Condition)				Main Effect (Generation)				Main Effect (Condition)			
Variables	Condition	Younger Adults (*n* = 20)	Older Adults (*n* = 34)	*F*	*p*-Value	η^2^	Observed Power	*F*	*p*-Value	η^2^	Observed Power	*F*	*p*-Value	η^2^	Observed Power
X-axis trajectory length (mm)															
	Control	−0.45 [−2.22–0.58]	−0.82 [−2.10–1.08]	1.035	0.359	0.020	0.227	0.866	0.356	0.016	0.150	1.691	0.189	0.031	0.349
	VR 30°	2.02 [0.04–5.85]	0.23 [−2.49–5.00]												
	VR 60°	2.25 [−0.26–4.88]	0.73 [−3.07–4.77]												
Y-axis trajectory length (mm)															
	Control	2.05 [−5.20–5.33]	0.85 [−0.88–3.70]	**3.436**	**0.036**	**0.062**	**0.633**	**4.210**	**0.045**	**0.075**	**0.522**	**8.346**	**<0.001**	**0.138**	**0.959**
	VR 30°	−4.15 [−7.66–1.03]	−0.58 [−5.43–3.23]												
	VR 60°	−5.58 [−7.12–−1.14] *	1.08 [−4.08–3.91] ^‡^												
Total trajectory length (mm)															
	Control	86.92 [77.07–96.00]	109.43 [91.68–142.88] ^‡^	**7.878**	**<0.001**	**0.132**	**0.936**	**33.724**	**<0.001**	**0.393**	**1.000**	**8.809**	**<0.001**	**0.145**	**0.959**
	VR 30°	83.55 [72.73–93.85]	157.45 [125.53–249.02] ^†,‡^												
	VR 60°	78.45 [65.10–107.06]	188.85 [112.75–283.36] ^†,‡^												
Trajectory length per unit time (mm/s)															
	Control	8.77 [7.82–9.69]	11.40 [9.36–14.57]	2.337	0.102	0.043	0.464	**13.897**	**<0.001**	**0.211**	**0.955**	**5.275**	**0.007**	**0.092**	**0.825**
	VR 30°	8.43 [7.34–9.48]	15.90 [12.67–25.17]												
	VR 60°	8.65 [6.91–10.79]	19.08 [11.39–28.63]												
Outer peripheral area (mm^2^)															
	Control	90.62 [65.39–114.62]	112.10 [73.80–147.38]	0.305	0.738	0.006	0.097	**6.960**	**0.011**	**0.118**	**0.735**	0.999	0.372	0.019	0.220
	VR 30°	87.40 [64.65–102.98]	124.63 [87.95–244.08]												
	VR 60°	71.98 [54.08–124.83]	146.47 [75.05–320.21]												
Rectangular area (mm^2^)															
	Control	150.82 [97.92–196.40]	174.92 [95.18–271.08]	0.115	0.892	0.002	0.067	**4.531**	**0.038**	**0.080**	**0.551**	0.779	0.462	0.015	0.180
	VR 30°	131.70 [88.69–201.73]	191.53 [136.54–417.10]												
	VR 60°	110.90 [68.23–254.09]	230.30 [108.96–616.88]												

Medians [interquartile range], η^2^: Eta-squared, VR Virtual Reality, * post-hoc test (*p* < 0.05) compared to control in younger adults, ^†^ post-hoc test (*p* < 0.05) compared to control in older adults, ^‡^ post-hoc test (*p* < 0.05) between younger and older adults.

**Table 3:** Results of COP movement in younger and older adult men and women.

		Younger Adults (*n* = 20)		Older Adults (*n* = 34)		*p*-Value				Effect Size			
		Men (*n* = 10)	Women (*n* = 10)	Men (*n* = 10)	Women (*n* = 24)	Men vs. Women		Younger vs. Older Adults		Men vs. Women		Younger vs. Older Adults	
Variables	Condition					Younger Adults	Older Adults	Men	Women	Younger Adults	Older Adults	Men	Women
X-axis trajectory length (mm)													
	Control	−0.40 [−1.96–0.85]	−1.00 [−3.61–0.87]	−1.62 [−2.28–0.06]	−0.32 [−1.88–2.02]	0.684	0.223	0.436	0.401	−0.102	0.211	−0.186	0.149
	VR 30°	0.35 [−0.67–4.68]	3.75 [1.98–7.05]	6.15 [0.51–10.68]	−1.40 [−2.94–3.56]	**0.043**	**0.001**	0.105	**0.002**	0.448	−0.525	0.372	−0.506
	VR 60°	−0.15 [−2.11–2.45]	4.40 [1.90–6.51]	6.33 [1.05–8.91]	−1.60 [−3.68–1.92]	**0.015**	**0.001**	0.052	**0.001**	0.541	−0.538	0.439	−0.538
Y-axis trajectory length (mm)													
	Control	2.45 [−4.53–8.92]	0.80 [−5.96–5.82]	0.12 [−4.37–4.13]	0.85 [−0.62–3.55]	0.739	0.539	0.631	0.564	−0.076	0.107	−0.118	0.100
	VR 30°	−4.15 [−8.48–−1.33]	−4.32 [−7.67–1.94]	−3.60 [−7.24–1.19]	0.20 [−4.22–3.37]	0.796	0.127	0.912	0.101	−0.068	0.266	0.034	0.285
	VR 60°	−6.05 [−8.30–−1.30]	−4.15 [−6.37–−0.22]	−4.62 [−10.13–−1.01]	−0.25 [−3.16–7.53]	0.315	**0.010**	0.912	**0.023**	0.237	0.431	0.034	0.389
Total trajectory length (mm)													
	Control	80.78 [68.02–102.94]	88.40 [81.85–92.41]	113.82 [91.68–146.91]	109.43 [90.18–142.03]	0.579	0.724	**0.035**	**0.010**	−0.135	−0.065	0.473	0.434
	VR 30°	78.42 [67.18–92.94]	86.88 [76.58–106.32]	213.70 [127.26–257.52]	149.47 [115.21–231.66]	0.218	0.341	**<0.001**	**<0.001**	0.287	−0.169	0.777	0.609
	VR 60°	78.45 [61.80–116.46]	78.93 [67.17–105.98]	186.65 [135.10–305.17]	188.85 [110.42–270.24]	0.912	0.589	**0.002**	**<0.001**	0.034	0.164	0.676	0.642
Trajectory length per unit time (mm/s)													
	Control	8.15 [6.87–10.39]	8.92 [8.27–9.34]	11.50 [9.27–14.83]	11.40 [9.44–14.51]	0.579	0.897	**0.035**	**0.006**	0.135	−0.023	0.473	0.460
	VR 30°	7.93 [6.79–9.37]	8.75 [7.76–12.21]	21.58 [12.86–26.02]	15.08 [11.62–23.39]	0.190	0.341	**<0.001**	**0.004**	0.304	−0.169	0.777	0.483
	VR 60°	7.93 [6.23–11.75]	9.25 [6.99–10.69]	18.85 [13.64–30.83]	19.08 [11.16–27.31]	0.739	0.589	**0.002**	**<0.001**	0.076	0.097	0.676	0.629
Outer peripheral area (mm^2^)													
	Control	86.32 [54.64–151.33]	97.43 [74.33–111.21]	122.85 [78.07–165.11]	106.38 [73.80–142.38]	0.684	0.696	0.393	0.564	0.102	−0.071	0.203	0.104
	VR 30°	84.17 [44.59–120.88]	87.77 [69.32–107.84]	201.30 [84.18–338.66]	117.83 [90.45–157.96]	0.739	0.304	0.052	**0.046**	0.085	−0.181	0.439	0.344
	VR 60°	79.88 [49.79–126.79]	66.25 [54.43–119.81]	163.35 [108.34–380.23]	133.23 [69.94–295.14]	0.912	0.445	0.063	**0.013**	−0.034	−0.136	0.423	0.421
Rectangular area (mm^2^)													
	Control	139.63 [77.79–283.65]	159.65 [120.43–226.82]	199.15 [112.05–377.66]	161.03 [93.31–254.50]	0.684	0.467	0.393	0.838	0.102	−0.130	0.203	0.039
	VR 30°	131.70 [55.60–248.45]	137.88 [92.88–195.44]	325.05 [131.16–679.30]	179.70 [126.24–347.65]	1.000	0.270	0.075	0.183	0.000	−0.194	0.406	0.233
	VR 60°	122.32 [60.49–274.91]	95.35 [71.44–235.01]	256.40 [184.33–658.20]	214.18 [99.24–584.26]	1.000	0.515	0.105	0.055	−0.017	−0.117	0.372	0.331

Medians [interquartile range], η^2^: Partial eta-squared, VR Virtual Reality.

## Data Availability

The data that support the findings of this study are available from the corresponding authors (N.M. or Y.U.) upon reasonable request.
